# Silica-Based RO Membranes for Separation of Acidic Solution

**DOI:** 10.3390/membranes9080094

**Published:** 2019-08-01

**Authors:** Katsunori Ishii, Ayumi Ikeda, Toshichika Takeuchi, Junko Yoshiura, Mikihiro Nomura

**Affiliations:** 1 Department of Applied Chemistry, Shibaura Institute of Technology, 3-7-5 Toyosu, Koto-ku, Tokyo 135-8548, Japan; 2 National Institute of Advanced Industrial Science and Technology (AIST), 1-1-1 Higashi, Tsukuba, Ibaraki 305-8565, Japan

**Keywords:** silica membrane, counter diffusion CVD method, chemical vapor deposition, reverse osmosis, H_2_SO_4_ solution separation, acid stability, silica substrate, sol-gel method

## Abstract

The development of acid separation membranes is important. Silica-based reverse osmosis (RO) membranes for sulfuric acid (H_2_SO_4_) solution separation were developed by using a counter diffusion chemical vapor deposition (CVD) method. Diphenyldimethoxysilane (DPhDMOS) was used as a silica precursor. The deposited membrane showed the H_2_SO_4_ rejection of 81% with a total flux of 5.8 kg m^−2^ h^−1^ from the 10^−3^ mol L^−1^ of H_2_SO_4_. The γ-alumina substrate was damaged by the permeation of the H_2_SO_4_ solution. In order to improve acid stability, the silica substrates were developed. The acid stability was checked by the gas permeation tests after immersing in 1 mol L^−1^ of the H_2_SO_4_ solution for 24 h. The N_2_ permeance decreased by 11% with the acid treatment through the silica substrate, while the permeance decreased to 94% through the γ-alumina substrate. The flux and the rejection through the DPhDMOS-derived membrane on the silica substrate were stable in the 70 wt % H_2_SO_4_ solution.

## 1. Introduction

The effective concentration of sulfuric acid (H_2_SO_4_) solution is highly demanded for saving energy because H_2_SO_4_ solution is usually distilled under low pressure to operate at lower temperatures due to the properties of the materials for the distillation columns. One of the possible applications of the concentration of H_2_SO_4_ solution is the iodine-sulfur (IS) process [1,2,3,4,5,6,7,8] to produce hydrogen from water by using heat at 600–1000 °C. The other application of the H_2_SO_4_ separation is the waste solution and recovery for the mining industry [9].

The separation of the acid solution by using a membrane has been mainly investigated for acetic acid (AcOH) solutions for industrial applications [10,11,12,13,14], membrane reactors [15], and microorganisms [16,17]. Polymeric membranes such as polyvinyl alcohol (PVA) membranes [11,13,15] or commercialized polymer membranes [10,17] had been applied for AcOH separation. H_2_SO_4_ separation performances were investigated by using commercialized polymer membranes [18,19,20]. However, acid stability is limited to polymeric membranes. Thus, inorganic membranes such as zeolite membranes [12] or silica membranes [14] have been investigated for nanofiltration (NF) or pervaporation (PV) membranes.

**Figure 1:**
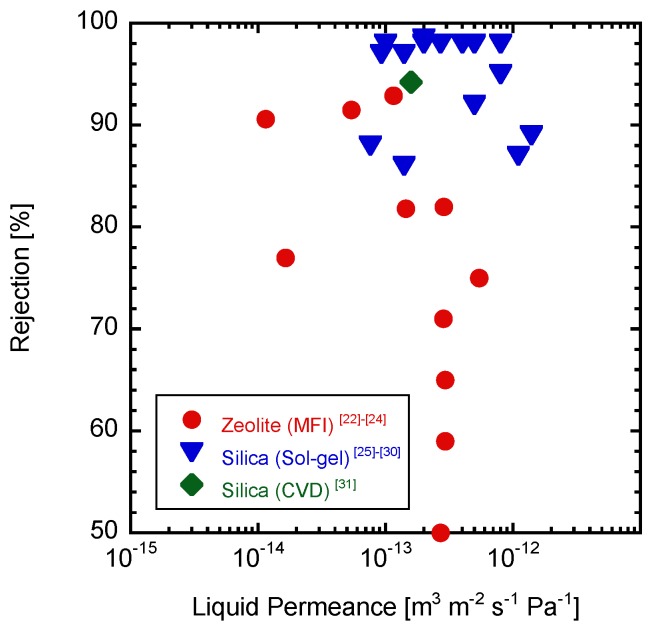
NaCl solution RO performance through the inorganic membranes.

Recently, inorganic RO (reverse osmosis) membranes such as zeolite [21,22,23,24] and silica membranes [25,26,27,28,29,30,31] have been reported. Figure 1 shows a summary of the sodium chloride (NaCl) RO performance through the inorganic membranes. The first report on inorganic membranes for RO application was the LTA (Linde Type A) zeolite membrane [21]. The ethanol rejection was 40% from 10 wt % of the aqueous ethanol solution. MFI membranes also have been investigated for RO permeation [22,23,24]. The Na^+^ rejection was 76.7% with the flux of 0.112 kg m^−2^ h^−1^ from the 0.1 M NaCl solution [22]. The flux through the MFI zeolite membranes could be improved by adding Al to the MFI structure [23].

**Figure 2:**
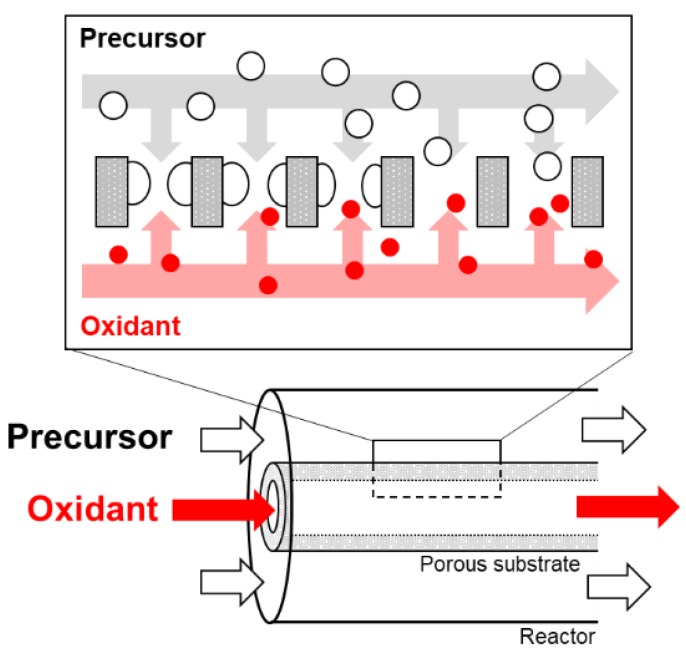
Counter diffusion chemical vapor diffusion method.

Silica membranes were also investigated for RO applications. Bis (triethoxysilyl) ethane (BTESE)-derived silica membranes prepared by using a sol-gel method showed a Na^+^ rejection of 95% with water permeability of 3 × 10^−3^ m^3^ m^−2^ s^−1^ Pa^−1^ [25]. The silica source of a cross-linked structure [27], having a hydroxyl group [29], or (polyhedral oligomeric silsesquioxane) POSS structure [30] is used. POSS and BTESE-derived silica membrane showed the Na^+^ rejection of 90% with a permeance of 1 × 10^−13^ m s^−1^ Pa^−1^ [30]. We have been developing silica membranes by using a chemical vapor deposition (CVD) method. Figure 2 shows a schematic diagram of the counter diffusion CVD method. silica precursor is supplied from one side of the porous substrate. An oxidant (O_2_ or O_3_) is supplied from the opposite side of the substrate. Deposition occurs in the pore of the substrate. Hydrogen permselective silica membranes were developed by using tetraethoxysilane or tetramethoxysilane as the silica precursors [32,33]. Recently, the pore size of the silica-based membranes was controlled by using organic-silicon alkoxides as the silica precursors [34]. The propyltrimethoxysilane-derived membrane showed a CH_4_/C_2_H_6_ permeance ratio of 20 [35]. The hexyltrimethoxysilane-derived membrane showed the C_3_H_6_/C_3_H_8_ permeance ratio of 414 [36]. The organic group of the silica precursor must work as a template of the deposited silica. Phenyltrimethoxysilane (PhTMOS)-derived membranes showed a Na^+^ rejection of 94.2% with a total flux 1.7 kg m^−2^ h^−1^ at 3.0 MPa [31]. However, the permeation performance and stability for the acid solution is not clear for the silica-based membranes prepared by using a counter diffusion CVD method.

In this study, a CVD silica-based membrane was applied to RO permeation of acid solution for a high concentration H_2_SO_4_ solution. PhTMOS and diphenyldimethoxyisilane (DPhDMOS) were examined as silica precursor. H_2_SO_4_ rejection and stability were investigated. Especially, the stability of the γ-alumina substrates and the improvement of the intermediate layer were discussed.

## 2. Materials and Methods

### 2.1. Porous Support

The porous ceramic substrate was consisted of two layers. One layer consisted of the porous α-alumina tube (NOK Co., Tokyo, Japan or Noritake Co. Ltd., Nagoya, Japan. With a pore size of 150 nm). The γ-alumina or silica layer were coated on the surface of the porous α-alumina tube. 

#### 2.1.1. γ-alumina Coating

The γ-alumina layer was prepared by the sol-gel method. The inside of the beaker glass was wetted by 3.84 g iso-propylalchoal (IPA, 99.7%, FUJIFILM Wako Pure Chemical Corporation, Osaka, Japan) in the glove box of the N_2_ atmosphere. 19.14 g of aluminum tri-sec-butoxyde (ALTSB, 97%, FUJIFILM Wako Pure Chemical Corporation, Osaka, Japan) was poured into the beaker and stirred slowly for 1 h to stabilize the interface between IPA and ALTSB. The ALTSB solution was removed from the glove box and moved to a separation funnel and was then dripped slowly to 72 mL of water at 90 °C and was stirred vigorously. The amount of water was kept even dripping the addition of ALTSB. The stirring was continued for 2 h until IPA was completely vaporized. Afterwards, the solution temperature was lowered to room temperature, and 28 mL of Nitric acid (HNO_3_, 1.0 mol L^−1^, FUJIFILM Wako Pure Chemical Corporation, Osaka, Japan) were added. The solution then refluxed for 12 h at 90 °C. The coating solution was prepared by mixing 30 mL of the refluxed solution, and 3.5 wt % polyvinylalchoal (n = 500, FUJIFILM Wako Pure Chemical Corporation, Osaka, Japan) then stirred for 10 min. This solution was used as a boehmite sol. All reagents were used without further purification.

The α-alumina ceramic tube was used as a support and dipped to boehmite sol for 30 s then calcined for 3 h at 600 °C. The dip-coating and calcination process were conducted two times to decrease the roughness of the γ-alumina layer. The coating support was called the γ-alumina substrate in this article. 

#### 2.1.2. Silica Coating

The silica layer was prepared by the sol-gel method. Tetraethoxysilane (TEOS, Shin-etsu Chemical Co., Ltd. Tokyo, Japan) and ethanol (EtOH, 99.5%, FUJIFILM Wako Pure Chemical Corporation, Osaka, Japan) was mixed and stirred in an ice bath for 10 min, then was dripped by HNO_3_ solution. Afterwards, the sol was stirred for 10 min in the ice bath and mixed for 1 h at room temperature. The molar ratio of the solution used was TEOS:EtOH:H_2_O:HNO_3_ = 1:5:4:0.1. All reagents were used without further purification.

The α-alumina tube ceramic was used as a support and dipped to silica sol for 60 s then calcined for 3 h at 600 °C. The dip-coating and calcination process were conducted two times to decrease the roughness of the silica layer. The coating support was called the silica substrate in this article.

### 2.2. Characterization

The prepared intermediate layers were characterized by scanning electron microscopy (SEM) imaging (KEYENCE, VE-8800, Osaka, Japan) and the pore size distribution was evaluated by nanopermporometry (Porometer nano, MicrotacBEL Corp., Osaka, Japan). The intermediate layer performance was characterized by a single gas permeation test and immersion of 1 mol L^−1^ H_2_SO_4_ solution for 24 h. The single gas permeances of H_2_, N_2_, and SF_6_ were measured by bubble flow meter at room temperature and at 100 °C. The gas permeance *P*_i_ [mol m^−2^ s^−1^ Pa^−1^] was calculated by Equation (1), where *n*_i_/*t* [mol s^−1^] shows the molecular permeation rate, *A* [m^2^] shows the membrane area, and *Δp* [Pa] shows the pressure difference of the membrane feed side and permeate side. The membrane selectivity was evaluated by the permeance ratio *α*_ij_ [-] using Equation (2).

(1) $$P_{i}\;=\frac{n_{i}}{t\;\cdot A\cdot \;\Delta p}$$

α_ij_ = *P*_i_/*P*_j_ (2)

### 2.3. CVD Procedures

**Figure 3:**
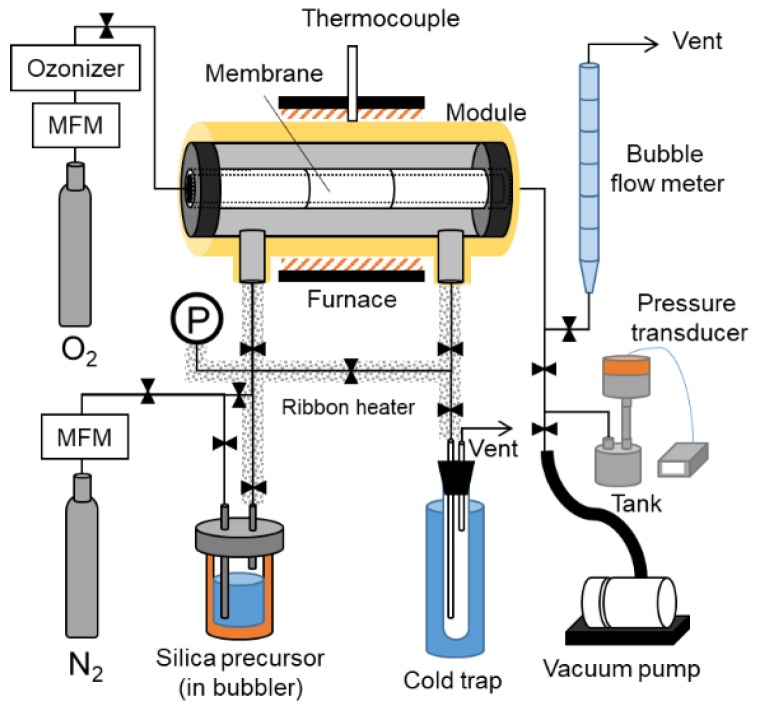
Counter diffusion CVD- gas permeation apparatus.

Figure 3 shows a schematic diagram of the counter diffusion CVD apparatus. The apparatus consists of a gas suppling part with a mass flow meter, ozonizer, and silica precursor in the bubbler, the reaction part of the module for the membrane, the vent part of the cold trap gas permeance measurement part with the bubble flow meter, the vacuum pump, and the pressure transducer.

The porous tuber substrate coated with γ-alumina or silica (Effective length: 30 mm; Effective area; 9.42 × 10^−4^ m^2^) was set in a membrane module with both ends of the substrate sealed by Viton O-rings. The substrate was heated by a furnace surrounding the membrane module to the deposition temperature in the N_2_ condition and kept to the deposition temperature. Ozone was produced by the ozone generator (ZOS-YB-6G, Shoken Co., Hiroshima, Japan) with an oxygen flow rate into the generator of 1.0 L min^−1^. Counter diffusion CVD was conducted when ozone was introduced into the membrane module at 0.2 L min^−1^ and through the inner part of the porous substrate. Simultaneously, the vaporized silica precursor (phenyltrimethoxysilane: PhTMOS, diphenyldimethoxyisilane: DPhDMOS, Shin-Etsu Chemical Co., Tokyo, Japan) in the bubbler was supplied to the outer side of the substrate by N_2_ bubbling with a flow rate of N_2_ which was 0.2 L min^−1^. The bubbler temperature was kept at 125 °C, and the deposition process was carried out at 230–400 °C for 90 min.

### 2.4. Membrane Characterization

The membrane performance was characterized by a single gas permeation test. Single gas permeances of H_2_, N_2_, and SF_6_ were measured by a bubble flow meter at room temperature and at 270 °C. The molecular sizes of the kinetic diameters are 0.29, 0.36, and 0.55 nm [35,37,38]. The membrane pore sizes were evaluated by the Normalized–Knudsen based permeance (NKP) method [39]. The pore sizes could be evaluated from single gas permeances by the fitting of the permeance and size of the permeated molecules. 

RO measurements were performed at room temperature. The H_2_SO_4_ feed solution concentration was kept at 10^−3^–10 mol L^−1^ and the feed pressure was kept at 4 MPa by using a liquid chromatography pump (L-6300, Hitachi High-Technologies Co., Ltd., Tokyo, Japan) or pH conductivity meter (CyberScan PC10, Eutech Instruments Pte. Ltd., Singapore). The H^+^ concentration was measured using an automatic titrator (COM-1700, HIRANUMA SANGYO Co., Ltd., Mito, Japan). The total flux of *J* [kg m^−2^ h^−1^] was calculated by Equation (3). *m* refers to the permeatated mass of liquid. *t* refers to the permeation time. *A* refers to the membrane area. The rejection of *R* was calculated using Equation (4) [40]. *C_p_* and *C_f_* refer to the concentrations of the permeate and feed solution.

The membrane stability was investigated by the RO test of 10^−3^ mol L^−1^ H_2_SO_4_ and immersion in 70 wt % H_2_SO_4_ was conducted twice.

(3) $$J=\frac{m}{t\cdot A}$$

(4) $$R=\left(1-\frac{C_{p}}{C_{f}}\right)\times 100\%$$

## 3. Results and Discussion

### 3.1. Pore Size Evaluation

**Figure 4:**
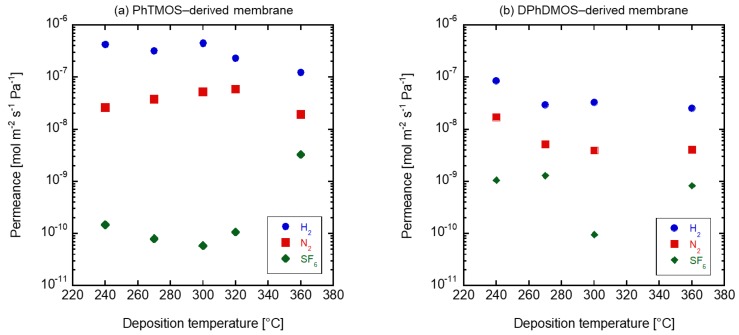
The gas permeation performance of (**a**) PhTMOS-derived and (**b**) DPhDMOS-derived membranes.

First, gas permeation tests were conducted to evaluate the pore sizes of the deposited membranes. Figure 4 shows the single gas permeation performance at room temperature through the (a) PhTMOS and (b) DPhDMOS-derived membranes by changing the deposition temperature. The permeance through the PhTMOS-derived membranes deposited between 240 and 320 °C showed a high N_2_/SF_6_ permeance ratio over 100. The highest N_2_/SF_6_ permeance ratio was 880 with N_2_ permeance of 2.3 × 10^−8^ mol m^−2^ s^−1^ Pa^−1^ through the membrane deposited at 300 °C. The N_2_/SF_6_ permeance ratio through the membrane deposited at 360 °C was low at 3.7. Ozone must be decomposed during the deposition not to deposit the silica. The DhPDMOS-derived membranes showed the lower N_2_/SF_6_ permeance ratio between 4 and 50 compared to those through the PhTMOS-derived membranes. The H_2_ permeances decreased with increasing the deposition temperature, and the deposition rate was slower than that for the PhTMOS deposition.

**Figure 5:**
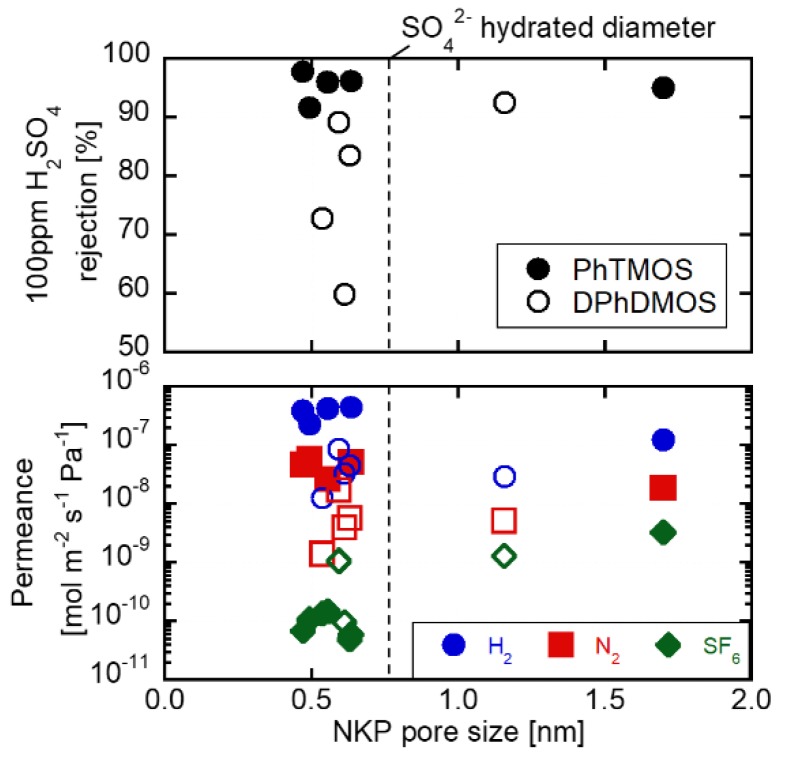
The NKP pore size of prepared membrane-derived gas permeance performance and H_2_SO_4_ rejection.

The pore sizes of the PhTMOS and DPhDMOS-derived membranes were evaluated by using the NKP method. Figure 5 shows the relationships of the pore sizes of the H_2_SO_4_ rejection and gas permeances through the PhTMOS and DPhDMOS-derived membranes. The pore sizes were between 0.47 and 1.70 nm. All membranes showed the pore sizes at ca. 0.5 nm with a higher N_2_/SF_6_ permeance ratio. The membranes have the potential of being applied in applications of hydrocarbon separation [35] and organic solvents separation [39]. Especially in acid solution RO tests, the PhTMOS and DPhDMOS membranes showed high rejection at 90% from the feed H_2_SO_4_ concentration of 10^−3^ mol L^−1^. The hydrated diameter of SO_4_^2^^−^ was 0.76 nm [41]. The pore size evaluation difference between the hydrated diameter and the NKP pore sizes requires further discussion.

The PhTMOS-derived membrane deposited at 360 °C showed the pore size at 1.70 nm, and the DPhDMOS-derived membrane deposited at 270 °C showed 1.15 nm. The phenyl groups on the silica surface decomposed at 360 °C [36]. These membranes showed a higher rejections over 90%, showing the effects of surface charge rejection. The zeta potential of silica is negative in 1.02 × 10^−3^ mol L^−1^ H_2_SO_4_ solution. 

### 3.2. H_2_SO_4_ Permeation through the Membranes Deposited on the γ-alumina Substrates

**Figure 6:**
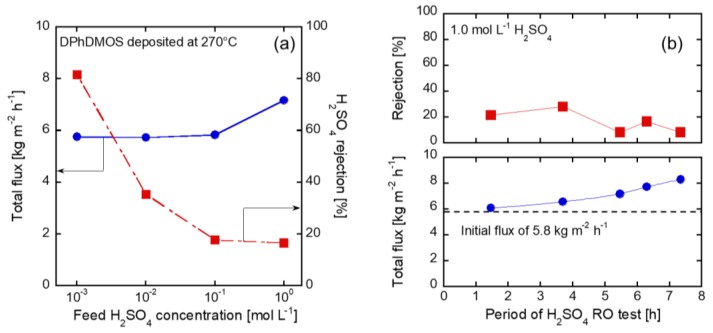
(**a**) Each H_2_SO_4_ concentration solution of the RO test, (**b**) the time course change of 1 mol L^−1^ H_2_SO_4_ RO test.

Figure 6a shows the RO performances through the PhTMOS and DPhDMOS-derived membranes from the feed concentration of 10^−3^ mol L^−1^. The rejections were over 90%. The flux through the PhTMOS and the DPhDMOS-derived membranes were 1.2 kg m^−2^ h^−1^ and 5.8 kg m^−2^ h^−1^, respectively. Both liquid and gas permeation through the DPhDMOS-derived membranes were higher than those through the PhTMOS membranes indicating that the loose silica structure was deposited from DPhDMOS. The chemical formula of PhTMOS consists of three methoxy groups, while there are only two methoxy groups for DPhDMOS. The amorphous silica network connection is formed from the alkoxy group of the silica precursors. Thus, the structure of the deposition from DPhDMOS was looser.

Figure 6a shows the effects of the feed H_2_SO_4_ concentration on the RO performance through the DPhDMOS-derived membrane. The fluxes, by changing the feed concentration from 10^−3^ to 10^−1^ mol L^−1^, were similar at 5.8 kg m^−2^ h^−1^. The rejections decreased by 18%, with increasing the feed H_2_SO_4_ concentration. The difference of the rejection could be explained by the degree of dissociation of H_2_SO_4_. The acid dissociation constant of H_2_SO_4_ and HSO_4_^−^ are 10^5^ and 10^−3^, respectively. Table 1 shows H_2_SO_4_ concentration, degree of dissociation, and osmosis pressure. Approximately 85% HSO_4_^−^ is ionized from the 10^−3^ mol L^−1^ solution. The rejection of 81% from the 10^−3^ mol L^−1^ solution could be explained by rejecting all the SO_4_^2−^. On the other hand, approximately 9% HSO_4_^−^ is ionized from the 0.1 mol L^−1^ solution. The rejection difference is affected by the change of ionized sulfate and hydration diameter and the membrane surface charge. The osmotic pressures increase with increasing H_2_SO_4_ concentration. The differences of the operation pressure and the osmotic pressure was 4.0, 3.94, 3.48 MPa. Lp was constant ca. 4.0 × 10^−13^ m^3^ m^−2^ s^−1^ Pa^−1^ from each H_2_SO_4_ solution at 10^−3^, 10^−2^, 10^−1^ mol L^−1^. The flux increased to 7.2 kg m^−2^ h^−1^ from the feed concentration of 1 mol L^−1^, the rejection was 17%. 

Figure 6b shows the time course of the fluxes and rejections from the 1 mol L^−1^ of the H_2_SO_4_ solution. The flux increased by increasing the operation time by 40% to 7.3 h. The rejection decreased from 22% to 8.3%. The membrane was broken under the permeation of the 1 mol L^−1^ of H_2_SO_4_ solution. Therefore, in order to investigate the acid stability, the stability of the γ-alumina substrates are discussed in the following section. 

### 3.3. Silica Substrates

**Figure 7:**
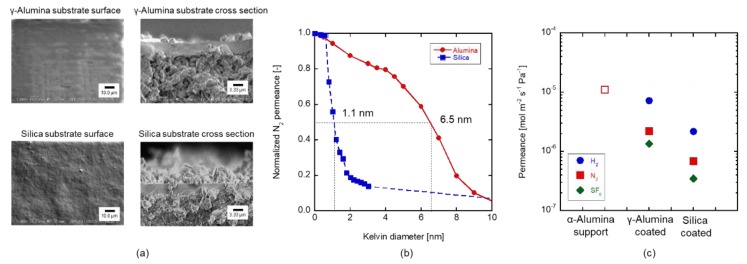
Characterization of the γ-alumina and silica substrate (**a**) the surface and cross-sectional view of the intermediate layer coated substrate, (**b**) the results of the nanopermporometer, and (**c**) the gas permeance.

In order to discuss the effects of the γ-alumina substrates, the silica layer was coated on the α-alumina tube. Figure 7a shows the surface and cross-section views of the SEM observation. Coating of the γ-alumina and silica layer was successfully carried out to obtain a uniform structure. The thickness of the intermediate layer of the γ-alumina and silica was 4.3 and 4.2 µm, respectively. Figure 7b shows a pore size distribution through the silica and the γ-alumina substrates by using the Kelvin condensation measurements. The average pore size of the silica substrate was about 1.1 nm, while that of the γ-alumina substrate was 6.5 nm. Figure 7c shows the gas permeances through the α-alumina tube, the silica substrate, and the γ-alumina substrate. N_2_ permeance through the silica substrate was 3.4 × 10^−7^ mol m^−2^ s^−1^ Pa^−1^ that is six times lower than that through the γ-alumina substrate. The low N_2_ permeance is explained by the smaller pore size of the silica substrate. The amount of sol that entered into the pore of the support was affected due to the particle size.

### 3.4. Acid Stability of the Substrates

**Figure 8:**
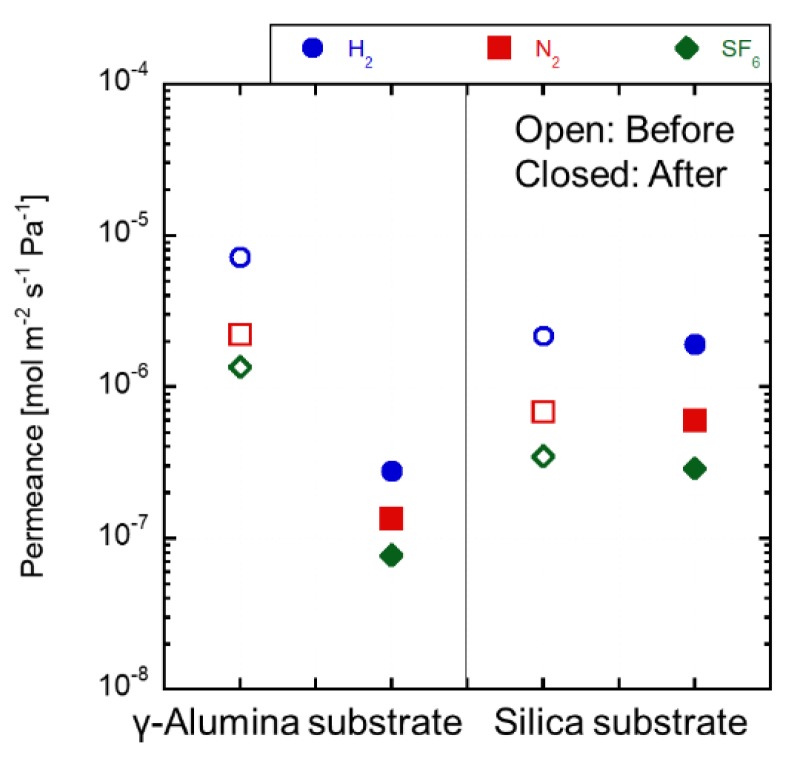
Gas permeance change of each substrate of 1 mol L^−1^ H_2_SO_4_ immersion before and after 24 h.

Figure 8 shows the gas permeances at 100 °C before and after the immersion tests. The N_2_ permeance through the as-made γ-alumina substrate was 2.2 × 10^−5^ mol m^−2^ s^−1^ Pa^−1^. The gas permeance after the immersion test decreased by approximately 10 times lower than that through the as-made substrate. The γ-alumina substrate was dissolved in the H_2_SO_4_ solution, and the dissolved alumina was filled in the pores of the γ-alumina substrate. The fluxes from the solution shown in Figure 8 increased. These phenomena could be explained by the γ-alumina layer dissolution for H_2_SO_4_ solution during the permeation tests. On the contrary, the gas permeance through the silica substrate kept constant by the immersion test. The permeance differences were within 11%. The silica substrate was stable in the 1.0 mol L^−1^ of the solution. 

### 3.5. CVD Treatment on the Silica Substrates

**Figure 9:**
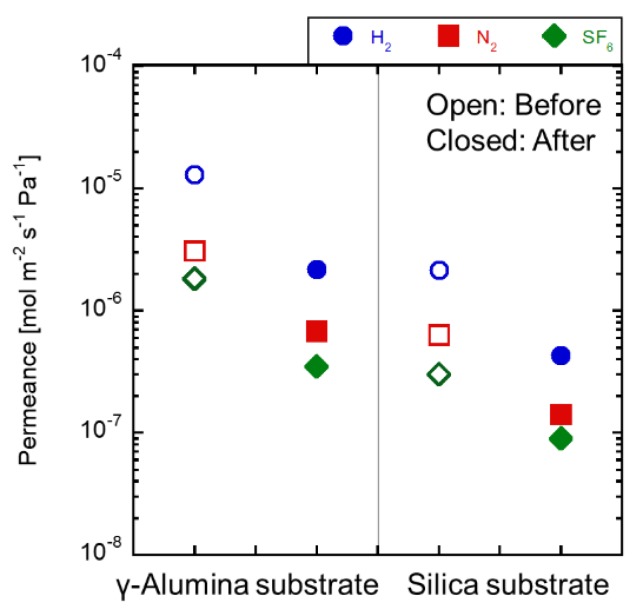
Gas permeance change of each substrate before and after CVD (Precursor: DPhDMOS, Deposition period: 90 min).

Figure 9 shows the gas permeances through the DPhDMOS-derived membranes prepared on the γ-alumina or silica substrate at 270 °C. The gas permeances were slightly decreased by the deposition. Both of the membranes gas permeance ratio showed the values of the approximate Knudsen ratio of H_2_/N_2_ = 3.7, N_2_/SF_6_ = 2.2. The pore sizes of both membranes could not be evaluated by the NKP method. The amount of deposited silica seems to have been small for both types of substrate. The deposition reaction occurs by gas diffusion of the substrate and the adsorption of the reactant, so further research is necessary for the reactivity of the deposition to the intermediate layer material and pore size.

### 3.6. RO Tests through the Membrane on the Silica Substrates

**Figure 10:**
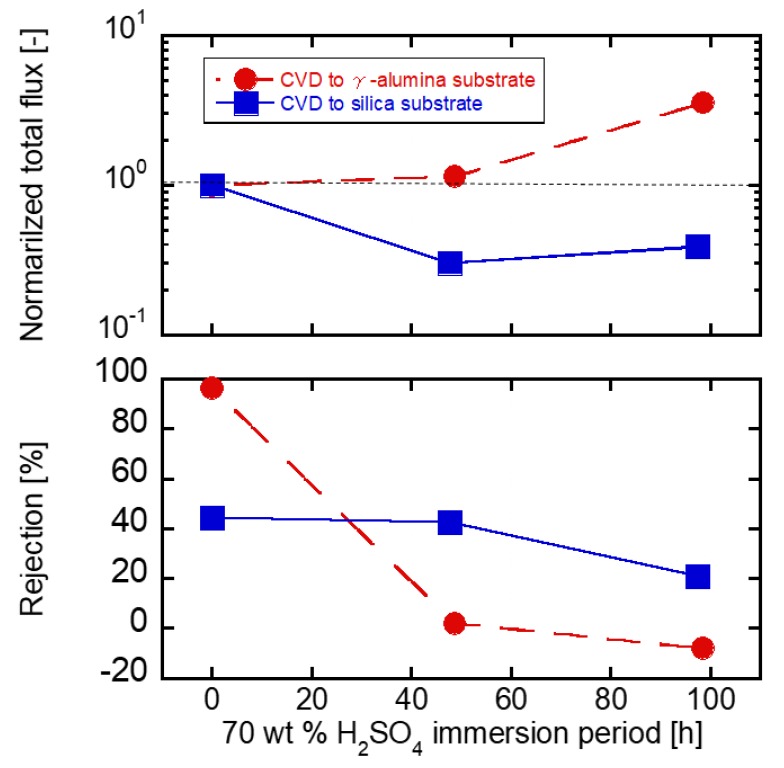
10^−3^ mol L^−1^ H_2_SO_4_ RO properties of CVD silica membrane.

Figure 10 shows RO performances through the DPhDMOS membrane prepared on the γ-alumina or the silica substrate. The as-made membrane on the γ-alumina substrate showed 96.3% of the rejection. After 48 h of the immersion procedure, the rejection deceased to only 2%. After 98 h of the immersion procedure, the flux increased three-fold than that through the as-made membrane. On the other hand, the rejection was 44% through the as-made membrane on the silica substrate n. However, after 48 h of the immersion procedure, the rejection was almost the same at 42%. The rejection was maintained after the immersion procedure in the 70 wt % H_2_SO_4_ solution. After 98 h of the immersion procedure, the rejection decreased to 21% while the flux increased slightly. The membrane of the silica substrate slightly dissolved by 70 wt % H_2_SO_4_. The acid stability was improved by preparing the membrane on the silica substrate.

## 4. Conclusions

The CVD silica membranes for the H_2_SO_4_ separation were developed. The DPhDMOS-derived membrane showed the flux of 5.7 kg m^−2^ h^−1^ with the rejection of 81.6% from the feed concentration of 10^−3^ mol ^−1^. The stability was not enough in the 1.0 mol L^−1^ of the H_2_SO_4_ solution due to the damage of the γ-alumina substrate. The γ-alumina layer was dissolved in the H_2_SO_4_ solution. Silica substrates were newly developed for the CVD membranes by using a sol-gel method. The pore size of the silica layer was 1.1 nm. The silica substrate was stable under 1 mol L^−1^ of the H_2_SO_4_ solution. The DPhDMOS-derived membrane deposited on the silica substrate was improved the stability under the 70 wt % of the H_2_SO_4_ solution.

## Figures and Tables

**Table 1:** Ion species abundance and osmotic pressure.

c [mol L^−1^]	α_2_	[SO_4_^2−^]	[HSO_4_^−^]	[H^+^]	Total Ionized Concentration [mol L^−1^]	π [MPa]	*Δp* [MPa]
10^−3^	0.847	0.000847	0.0002	0.00185	0.002847	0.007	3.9929
10^−2^	0.418	0.004183	0.0058	0.01418	0.024183	0.060	3.9400
10^−1^	0.086	0.008587	0.0914	0.10859	0.208587	0.517	3.4829
10^0^	0.010	0.009998	0.9900	1.01000	2.009998	4.983	−0.9827

c: H_2_SO_4_ concentration, α_2_: Secondly acid dissociation constant, [SO_4_^2−^]: Abundance concentration of SO_4_^2−^ to prepared solution, [HSO_4_^−^]: Abundance concentration of HSO_4_^−^ to prepared solution, [H^+^] Abundance concentration of H^+^ to prepared solution, π: osmotic pressure, *Δp*: Intermembrane pressure difference.
